# Reducing meat consumption by appealing to animal welfare: protocol for a meta-analysis and theoretical review

**DOI:** 10.1186/s13643-019-1264-5

**Published:** 2020-01-06

**Authors:** Maya B. Mathur, Thomas N. Robinson, David B. Reichling, Christopher D. Gardner, Janice Nadler, Paul A. Bain, Jacob Peacock

**Affiliations:** 1 Quantitative Sciences Unit, Stanford University, Stanford, USA; 2 Stanford Solutions Science Lab, Department of Pediatrics, Stanford University, Stanford, USA; 3 Department of Oral and Maxillofacial Surgery, University of California at San Francisco, San Francisco, USA; 4 Stanford Prevention Research Center, Stanford University, Stanford, USA; 5 American Bar Foundation, Chicago, USA; 6 Pritzker School of Law, Northwestern University, Chicago, USA; 7 Countway Library of Medicine, Harvard University, Cambridge, USA; 8 The Humane League Labs, Rockville, USA

**Keywords:** Behavior change, Nutrition, Meat consumption, Meat paradox, Animal welfare

## Abstract

**Background:**

Reducing meat consumption may improve human health, curb environmental damage and greenhouse gas emissions, and limit the large-scale suffering of animals raised in factory farms. Previous work has begun to develop interventions to reduce individual meat consumption, often by appealing directly to individual health motivations. However, research on nutritional behavior change suggests that interventions additionally linking behavior to ethical values, identity formation, and existing social movements may be particularly effective and longer-lasting. Regarding meat consumption, preliminary evidence and psychological theory suggest that appeals related to animal welfare may have considerable potential to effectively leverage these elements of human psychology. We aim to conduct a systematic review and quantitative meta-analysis evaluating the effectiveness of animal welfare-related appeals on actual or intended meat consumption or purchasing. Our investigation will critically synthesize the current state of knowledge regarding psychological mechanisms of intervening on individual meat consumption and empirically identify the psychological characteristics underlying the most effective animal welfare-based interventions.

**Methods:**

We will systematically search eight academic databases and extensively search unpublished grey literature. We will include studies that assess interventions intended to reduce meat consumption or purchase through the mention or portrayal of animal welfare, that measure outcomes related to meat consumption or purchase, and that have a control condition. Eligible studies may recruit from any human population, be written in any language, and be published or released any time. We will meta-analyze the studies, reporting the pooled point estimate and additional metrics that describe the distribution of potentially heterogeneous effects. We will assess studies’ risk of bias and conduct sensitivity analyses for publication bias. We describe possible follow-up analyses to investigate hypothesized moderators of intervention effectiveness.

**Discussion:**

The findings of the proposed systematic review and meta-analysis, including any identified methodological limitations of the existing literature, could inform the design of successful evidence-based interventions with broad potential to improve human, animal, and environmental well-being.

**Systematic review registration:**

The protocol was preregistered via the Open Science Framework (https://osf.io/d3y56/registrations).

## Background

Excessive consumption of meat and animal products may be deleterious to human health (with meta-analytic evidence regarding cancer [1–4], cardiovascular disease [5–7], metabolic disease [8–10], obesity [11], stroke [12], and all-cause mortality [13], albeit sometimes subject to methodological limitations), promotes the emergence and spread of antibiotic-resistant pathogens [14], is a major source of greenhouse gas emissions and environmental degradation [15], and contributes to market demand for industry practices that cause the preventable suffering of more than one billion animals in the USA annually [16]. Therefore, developing simple, effective interventions to reduce meat consumption could carry widespread societal benefits. Direct appeals regarding individual health may be effective [17], but comparatively little attention has been devoted to appeals regarding animal welfare [17]. However, the nascent literature on the psychology of meat consumption suggests that animal welfare appeals might be particularly promising [18]. Whereas behavior-change appeals emphasizing individual health sometimes suffer from high recidivism [19, 20], interventions that instead link behavior to ethical values, self-identity, and existing social movements may be especially potent and long-lasting [20–22]. Interventions of this type, sometimes termed “stealth interventions,” have for example successfully reduced childhood obesity-related behaviors and risk factors for cardiovascular disease and diabetes by appealing to cultural and ethical values in order to increase physical activity, rather than by appealing directly to obesity reduction or other health benefits [23–25].

Ethical concern about farm animal suffering is now a majority stance in several developed countries [26], yet meat consumption remains nearly universal (the “meat paradox” [27]). How does meat-eating behavior survive the resulting cognitive dissonance? Individuals may, post hoc, reduce their attributions of mind and sentience to meat animals, limiting the moral relevance of eating meat. For example, subjects randomly assigned to eat beef subsequently reported that cows are less capable of suffering, and they showed less moral concern, than subjects randomly assigned to eat nuts [28]. Simple appeals encouraging mind attribution to meat animals may disarm this dissonance-reduction strategy, thus reducing willingness to eat meat [29–31]. Additionally, physical disgust and moral disgust are closely intertwined. Experiencing physical disgust can amplify negative moral judgments, even when the two sources of disgust are conceptually unrelated (e.g., viewing physically disgusting video clips versus judging cheating behavior); conversely, experiencing moral disgust can induce physical disgust [32]. Given the powerful impact of physical disgust on food choices [33], evoking moral disgust regarding animal suffering may be a particularly potent means of shaping food choices [34]. Furthermore, many appeals regarding animal suffering are themselves physically disgusting (e.g., by depicting conditions in factory farms), perhaps further leveraging the moral-physical disgust connection.

Piggybacking on an existing, more broadly based social movement regarding farm animal welfare may further enhance the impacts of such interventions by triggering additional “process motivations” for participation [20, 21]. That is, the process of participating in a social movement (e.g., reducing meat consumption due to ethical concerns) may be motivating in itself, above and beyond the motivation provided by the ultimate outcomes of participation (e.g., improved animal welfare). In addition to providing opportunities for self-, group-, and social-identity development, participation in a social movement may also provide opportunities for social interaction, perceived belonging, social support, behavioral modeling, and activities that boost perceptions of collective efficacy [35]. This may reduce the risks of perceived individual failure and associated threats to self-efficacy [35] by defining participation in the movement itself as a success [20, 21]. The existence of a broader social movement may additionally trigger group- or societal-level changes (e.g., increased public attention, shifting norms regarding meat consumption, or decreased availability or increased prices of meat) that may alter the social and physical environments to make it easier to sustain the new behaviors [20, 21].

Recognition of this rich social, moral, and affective psychology underlying meat consumption [36, 37] has led to some promising, theoretically motivated academic work on new intervention strategies employing animal welfare-based appeals. In addition to that academic literature, evidence-based nonprofits have also started to produce some well-conducted, pragmatic experiments that have been reported in a separate body of “grey” literature. Through an interdisciplinary collaboration whose combined expertise spans nutrition, psychology, nonprofit research, statistics, and systematic reviews, we will conduct a systematic review and meta-analysis to address the primary research question: “How effective are animal welfare appeals at reducing consumption of meat or animal products?” We define “meat” as edible animal flesh and “animal products” as other edible products harvested from animals, such as eggs and milk. As a second objective, we will evaluate the risks of bias in this literature. As a third objective, we will discuss theoretical advances underlying effective interventions based on animal welfare appeals, highlighting any interesting connections between existing theories, which have often been pursued in isolation, and suggesting potential future directions for experimental psychology research.

## Systematic review methods

### Eligibility criteria

Eligible studies will satisfy the following criteria:

**Population:** Studies recruiting subjects from any human population, including online crowdsourcing websites, are eligible.

**Intervention:** Our review will consider animal welfare appeals to reduce meat consumption or purchase. Specifically, the intervention must be intended to reduce meat consumption or purchase and must include any explicit or implicit mention or portrayal of farmed animals, their suffering, their slaughter, or their welfare. Interventions need not specifically mention meat consumption or purchase. Interventions must directly depict or refer to farmed animals or items directly related to their welfare in any graphical, visual, textual, or auditory format. Any format and duration of the intervention is eligible. Composite interventions including both an animal welfare appeal and some other form of appeal (e.g., environmental) are eligible^1^ .

**Comparison:** The study must include a control group, condition, or time period not subjected to any form of intervention intended to change meat consumption (e.g., a two-group study comparing an animal welfare appeal to an environmental appeal also intended to reduce meat consumption would be ineligible). Not exhaustively, eligible study designs therefore include randomized controlled trials, repeated-measures designs in which subjects serve as their own controls, and quasi-experiments such as interrupted time-series analyses. For example, a repeated-measures study might estimate the difference in subjects’ meat consumption after versus before all subjects are exposed to an eligible intervention. In a quasi-experiment, posters encouraging reduced meat consumption might be introduced to a cafeteria on a particular day, with meat consumption measured before and after this intervention.

**Outcome:** Our review will consider meat consumption or purchase as outcomes. Specifically, the outcome must be actual (i.e., behaviorally measured rather than reported), self-reported, or intended consumption or purchase of meat or animal products. Willingness to pay for these products is an eligible outcome, though we anticipate that most such studies will include only comparisons of different meat products without a non-meat option, hence failing the previous inclusion criterion requiring a control condition. Studies assessing these outcomes in virtual or lab environments are eligible. Studies measuring only a specific type of meat or animal product (e.g., chicken) are eligible. Outcomes such as empathy, affective responses to meat, beliefs about factory farming, etc., are ineligible, though these outcomes may be relevant for narrative discussion in the theoretical review. Table 1 provides examples of eligible outcome measures by category and method of measurement (actual, self-reported, or intended).

**Table 1:** Examples of eligible outcome measures by category (consumption or purchase) and method of measurement

Measurement	Meat consumption	Meat purchase
Actual	Amount of meat (e.g., by weight) that subject self-serves at a buffet	Total cost of meat purchases listed on subject’s last grocery-store receipt
Self-reported	Amount of meat that subject reports having eaten during the past week	Amount of money that subject reports having spent on meat during the past week
Intended	Amount of meat that subject reports intending to eat during the next week	Reported willingness to pay for a meat versus non-meat dish

**Time frame:** Articles released or published at any point prior to our final search are eligible.

### Search methods

In stage 1 searches, we will search both the peer-reviewed literature and the grey literature to identify eligible articles. Then, in stage 2 searches, we will use “snowballing” of the articles identified in stage 1 to attempt to identify additional articles, as described below. We will include articles and grey literature published or released at any time and written in any language, seeking translation for full texts if necessary.

#### Stage 1 searches

##### Journal articles and academic grey literature

We developed a sensitive search strategy for peer-reviewed literature in collaboration with an academic reference librarian (PAB). We will search eight databases (Medline, Embase, Web of Science, PsycInfo, CAB Abstracts, Sociological Abstracts, ProQuest Dissertations & Theses, and PolicyFile) using search terms in Additional file 1, Section 2. A pilot search using these terms retrieved 3779 articles after removing duplicates.

##### Non-academic grey literature

An author (JP) who is the director of an evidence-based animal welfare research organization will lead the search for non-academic grey literature. We will identify relevant studies conducted or referenced by evidence-based animal welfare organizations by searching, not exhaustively, the websites of Faunalytics, Animal Welfare Action Lab, The Humane League Labs, Mercy for Animals, Animal Charity Evaluators, and Better Buying Lab (Additional file 1, Section 3). Additionally, JP will use his professional knowledge and personal contacts to identify other unpublished studies.

#### Stage 2 searches

In Stage 2 searches, we will attempt to find missed articles by reviewing the reference lists of eligible and closely relevant articles identified during Stage 1. We will also consider sending a survey to corresponding authors of such studies and researchers at top animal welfare organizations to solicit leads to additional published or unpublished studies that may be eligible.

### Article review methods

We will review articles and manage data using the software applications Covidence [38] and Microsoft Excel. Each article retrieved from an academic database in Stage 1 will first undergo title/abstract screening by at least two reviewers. In this stage, reviewers will exclude articles that clearly fail the inclusion criteria. Remaining articles will proceed to a full-text screening, during which at least two reviewers will specifically assess each inclusion criterion. We will resolve conflicts between reviewers through discussion or adjudication by other authors. For articles identified through manual searches of grey literature or the manual processes in Stage 2, the author on our team who identified the article will assess whether the article clearly fails inclusion criteria, conservatively retaining articles for further review that might satisfy inclusion criteria. These remaining articles will be entered in Covidence for full-text screening. We henceforth refer to articles ultimately judged to meet all inclusion criteria as “eligible.”

### Data extraction methods

For each eligible article that reports sufficient statistical information, the statistician (MBM) will extract point estimate(s) and variance estimate(s) most closely approximating the treatment effect of the animal welfare appeal (see Additional file 1, Section 4 for details). When relevant statistics are not reported, we will hand-calculate them from available statistics, plots, or publicly available datasets as feasible, or will contact study authors. Articles reporting multiple point estimates on separate subject samples or on samples sharing a control group may contribute all of these point estimates to the analyses; the “Qualitative and quantitative analyses” section discusses statistical assumptions regarding independence and alternative models to be used if independence assumptions may be violated.

Articles reporting multiple point estimates on entirely overlapping samples (e.g., from a study measuring multiple eligible outcomes) may similarly contribute one point estimate per eligible intervention and outcome because different point estimates may be relevant to different moderator analyses described in the “Qualitative and quantitative analyses” section. However, point estimates on entirely overlapping samples will not be included together in the same meta-analysis. Similarly, if multiple articles report on the same study, we will include the point estimate from only the study with the largest sample size. For the main, overall analysis, we will designate a primary point estimate for each study with multiple eligible point estimates on the same subject sample as follows. With the following criteria considered in order, interventions will be given precedence when they are specific to animal welfare (e.g., they do not include other types of appeals), when they have the most detailed content, and when they have the longest duration. Some studies may report consumption of all meat or all animal products as well as of specific types of meats, in which case the outcome that most closely matches the intended scope of the intervention will be given precedence (e.g., the outcome “beef consumption” for an intervention specifically targeting beef consumption; the outcome “all-meat consumption” for an intervention targeting all-meat consumption). As described in the “Qualitative and quantitative analyses” section, we also plan to conduct sensitivity analyses excluding interventions that target consumption of a specific type of meat, rather than the consumption of all meats. For longitudinal studies, outcomes measured with the longest follow-up will be given precedence.

## Qualitative and quantitative analyses

### Qualitative review

We will use a table to describe study characteristics, such as the study’s randomization status, the specific intervention and outcome (including which specific meats or animal products were included in the outcome measure), the length of follow-up between the intervention and outcome measurement, subject demographics (e.g., location, percent of male subjects, mean age), and the analyzed sample size. We will also descriptively present a table of risk-of-bias criteria (e.g., Table 2). To develop our risk of bias tool, we adapted from the Cochrane Risk of Bias Tool for randomized studies [39] those criteria that are relevant to the literature we intend to synthesize (e.g., criteria related to selection bias, attrition bias, and reporting bias), removed some criteria that are not relevant or feasible for this literature (e.g., regarding blinding), and expanded the generic category “Other bias” into criteria of particular relevance to this literature (e.g., regarding the quality of outcome measurement and the potential for social desirability bias). We adapted the existing items in order, for example, to allow assessment of the stated type of bias in the diverse study designs that we expected to encounter. To develop the tool, a group of authors (MBM, JP, DBR, JN) read studies known to meet eligibility criteria and, based on their combined expertise in meta-analysis methodology and in research on interventions to reduce meat consumption, developed the risk of bias tool through discussion, piloting on eligible studies, and iterative revision.

**Table 2:** Criteria for risk of bias

Criterion	Example of low risk of bias	Example of high risk of bias
Exchangeability of the control and intervention groups	The study is randomized.	The study is observational with uncontrolled self-selection into the intervention group (e.g., inducing confounding by a pre-existing interest in dietary change).
Proximity of the outcome measure to actual meat consumption or purchase	The study measures meat consumption using subjects’ actual food choices in a cafeteria.	The study measures subjects’ intended meat consumption.
Missing data	Nearly all enrolled subjects completed the intervention and provided outcome measures.	Many subjects failed to complete the intervention or were lost to follow-up before the outcome was measured.
Minimization of social desirability biases and demand characteristics	The intervention was subtly embedded in a decoy task about a topic unrelated to meat consumption, leading subjects to believe the study was not about meat consumption.	Subjects interact with experimenters who are clearly identifiable as animal welfare advocates.
Potential for selective reporting	The study was preregistered.	The study was not preregistered
Analytic reproducibility	The study has publicly available data, materials, and code.	The study does not have publicly available data, materials, or code.

For the narrative review of psychological theory underlying the interventions, we will read eligible articles in depth to identify categories of proposed mechanisms or theoretical motivations (e.g., attribution of sentience to meat animals). We will discuss the state of theoretical development and open issues in each of these categories, paying particular attention to any longitudinal studies that conduct mediation analyses using measurements of proposed mechanistic variables (e.g., a subject’s estimate of a cow’s capacity to suffer) occurring after exposure to the intervention and before measurement of the outcome. We may also discuss articles that contribute useful theoretical insights but that are not eligible for inclusion in the quantitative meta-analyses, for example, because they measure proposed mechanistic variables without an eligible outcome measure of meat consumption or purchase.

### Quantitative meta-analyses

#### Primary analyses and sensitivity analyses

Pilot searches indicate that quantitative meta-analysis will be feasible. These searches indicated that at least 20 published and unpublished papers, totaling at least 50 point estimates, will meet inclusion criteria. Additionally, interventions to date have many common elements of design and delivery, such that a random-effects estimate of their mean treatment effect should have a meaningful interpretation. The statistician (MBM) will conduct all statistical analyses in R [40]. We will extract point estimates on the risk ratio scale (see Additional file 1, Section 4 for details). We will fit random-effects meta-analysis models using restricted maximum likelihood (REML) via the R package *metafor*. If the point estimates appear non-normal or if many studies contribute multiple point estimates, potentially introducing correlation between point estimates, we will instead (or additionally) fit a robust semi-parametric meta-analysis model using the R package *robumeta* [41, 42]. Reporting will focus primarily on point estimates, 95% confidence intervals, and *p* values for the mean effect size and the estimated standard deviation of the effect sizes ($\hat{\tau}$). We will report *p* values as continuous metrics rather than relative to a dichotomous *α* threshold.

For the primary analysis, we will estimate the interventions’ average effect size by meta-analyzing the point estimates from all eligible studies that provided sufficient information to extract data. We have designated this analysis as primary because it represents a reasonable first step in pursuing the first quantitative meta-analysis of this nascent literature. The primary analysis will aggregate across all eligible outcomes (Table 1) rather than distinguish between main versus additional outcome measures because we anticipate that a large majority of studies will fall into only one or two categories of outcomes. However, as described in the “Secondary analyses” section below, we will conduct subset or moderator analyses by the outcome if feasible.

If the interventions’ effects appear heterogeneous based on the statistical estimate of their standard deviation ($\hat{\tau}$), we will characterize heterogeneity as follows. First, we will estimate the percentage of true effects that are stronger than various effect-size thresholds (e.g., risk ratios of 1, 1.1, and 1.2) [43, 44]. We will additionally estimate the percentage of true effects with risk ratios less than 1, representing unintended *detrimental* effects of the interventions [43, 44]. These metrics can help identify if (1) there are few effects of scientifically meaningful size despite a “statistically significant” pooled point estimate; (2) there are some large effects despite an apparently null point estimate; or (3) strong effects in the direction opposite of the pooled estimate also regularly occur [43]. Second, as a hypothesis-generating method to help identify the individual interventions that appear most effective, we will estimate the true effect size in each study using a nonparametric shrinkage method [45], qualitatively reviewing the characteristics of those interventions with the largest estimated true effect sizes.

We will conduct several sensitivity analyses regarding statistical biases and the scope of articles included in the analysis. First, we will assess publication bias using selection model methods [46], sensitivity analysis methods [47], and the significance funnel plot [47]. Given the relatively small anticipated number of eligible studies, we anticipate that selection models may fail to converge or may provide extremely wide, uninformative confidence intervals^2^, in which case we may omit them and present only the sensitivity analysis approach. As a further sensitivity analysis, we will exclude (1) composite interventions (e.g., appeals discussing both animal welfare and carbon emissions); (2) studies that were borderline with respect to the inclusion criteria [48]; and (3) interventions targeting consumption of only a specific type of meat (e.g., beef) rather than consumption of all meat, which might potentially shift consumption from one type of meat to another (e.g., chicken) without reducing total consumption. If statistical confounding appears to be a widespread problem, we will consider conducting relevant sensitivity analyses [49].

#### Secondary analyses

We will consider using meta-regression or subset analyses to assess the role of hypothesized moderators (e.g., Table 3), but we anticipate that sample sizes may be inadequate to yield precise conclusions using this approach. Additionally, we anticipate that most studies will report outcomes in terms of changes in servings of meat. We will conduct secondary analyses in which we instead express intervention effectiveness using metrics that more directly characterize societal impact, such as (1) the estimated reduction in human all-cause mortality events [50]; (2) the estimated reduction in the number of animals reared for consumption; and (3) the estimated reductions in greenhouse gas emissions based on estimates for different types of animals [51]. We will conduct separate meta-analyses using each of these three metrics.

**Table 3:** Hypothesized moderators for meta-regressive or subset analyses

Hypothesized moderator	Possible categories for analysis
Study design	Randomized vs. all other designs
Sex ratio of subjects	0–30% female vs. 30–70% female vs. 70–100% female
Psychological theory/theories underlying the intervention	Mind attribution vs. disgust vs. social norms, etc.
Intensiveness of intervention	Total length of time subjects are exposed to intervention
Length of follow-up between intervention and outcome measurement	Days (continuous) or *>* 1 day vs. *≤* 1 day
Proximity of the outcome measure to actual meat consumption or purchase	Behaviorally measured vs. self-reported vs. intended consumption or purchase
Scope of meat outcome	All-meat consumption vs. any subset of meats (where studies excluding seafood consumption will be in the latter category)

## Discussion

Developing effective interventions to reduce excessive meat consumption could carry broad societal benefits to human health, animal welfare, and the environment. Interventions involving appeals regarding animal welfare may have unique potential to transform widespread ethical concerns about farm animal suffering into actual behavioral change and to tap potent psychological levers, such as cognitive dissonance and the connection between physical and moral disgust. However, these types of interventions have received little academic attention to date. The present systematic review will discuss theoretical advances underlying effective interventions based on animal welfare appeals. By further conducting the first quantitative meta-analysis of animal welfare-based appeals to reduce meat consumption, we will estimate the effectiveness of the interventions that have been attempted to date, assess what characteristics of interventions may contribute to greater effectiveness, and present findings using metrics that directly summarize their societal impact on human health, animal welfare, and the environment. Last, by carefully assessing risks of bias of the existing literature, we will provide concrete methodological recommendations for future work.

## Supplementary information

**Additional file 1:** **Section 1.** Examples of eligible interventions. **Section 2.** Search terms by database. **Section 3.** Examples of websites to be searched for grey literature. **Section 4.** Details of data extraction.

## Data Availability

The manuscript will report and justify any deviations from the preregistration, and any analyses introduced post hoc will be delineated as such. All data, materials, and analysis code required to reproduce all results will be publicly released on the first author’s Open Science Framework or Github repositories immediately upon publication (or earlier if possible given journal policies).

## Abbreviation

REML: Restricted maximum likelihood

## Footnotes

1 As described in the section “Qualitative and quantitative analyses”, we will conduct sensitivity analyses excluding composite interventions. We use “sensitivity analysis” to refer to analyses designed to assess the extent to which the results of primary analyses are affected by different design or analysis choices (e.g., alternative inclusion criteria or different statistical model specifications) or to internal biases such as publication bias [48]. Examples of eligible interventions are provided in Additional file 1, Section 1.

2 Convergence failure means that a statistical method is unable to arrive at a numerical estimate, usually due to small sample sizes or extreme distributions of data. Wide confidence intervals reflect uncertainty about a point estimate and can occur when the data do not provide enough information for precise estimation.
