# Supplementary material for: Reducing meat consumption by appealing to animal welfare: protocol for a meta-analysis and theoretical review
Source: Syst Rev. 2020 Jan 6;9:3. doi: 10.1186/s13643-019-1264-5 (PMC6945605; doi:10.1186/s13643-019-1264-5)
Supplement: Supplementary file 1 — Additional file 1: Section 1. Examples of eligible interventions. Section 2. Search terms by database. Section 3. Examples of websites to be searched for grey literature. Section 4. Details of data extraction. [file 13643_2019_1264_MOESM1_ESM.pdf]

Additional File 1: Reducing Meat Consumption by Appealing to Animal Welfare

Contents

1 Examples of eligible interventions 1

2 Search terms by database 1

2.1 MEDLINE (Ovid) . . . 1
2.2 Embase (Elsevier) . . . 2
2.3 Web of Science (Clarivate Analytics) . . . 2
2.4 PsycINFO (EBSCO) . . . 2
2.5 CAB Abstracts (Clarivate Analytics) . . . 3
2.6 Sociological Abstracts (ProQuest) . . . 3
2.7 ProQuest Dissertations and Theses (ProQuest) . . . 3
2.8 PolicyFile (ProQuest) . . . 3

3 Examples of websites to be searched for grey literature 4

4 Details of data extraction 4

1. Examples of eligible interventions

Regarding explicit and implicit interventions, an eligible explicit intervention might involve calling subjects and asking them to reduce meat consumption in order to reduce animal suffering. An eligible implicit intervention might involve posting in a cafeteria photographs of animals reared in factory farms. Regarding direct depictions of or references to animal welfare, an intervention consisting only of a photograph of empty gestation crates would be eligible, but not an intervention consisting only of photographs of meat being processed in a physically disgusting manner. Possible examples of different formats and durations of interventions include durations of interventions: attendance in a class, receipt of daily text messages, receipt of a leaflet or mock news article, and exposure to a 10-minute video.

2. Search terms by database

2.1. MEDLINE (Ovid)

(animal welfare/ OR emotions/ OR disgust/ OR fear/ OR guilt/ OR empathy/ OR morals/ OR (emotion* OR affective OR disgust* OR fear OR guilt* OR empath* OR suffer* OR moral* OR ethic* OR humane* OR anthropomorph* OR belief* OR cognitive dissonan* OR meat paradox).ab,ti OR (animal* adj6 (welfare OR rights OR wellbeing OR well being

OR cruel* OR abuse OR mistreat*)).ab,ti) AND ((meat* OR animal product* OR beef OR veal OR lamb OR pork OR poultry OR chicken OR turkey OR pig OR cow OR sheep) adj6 (consum* OR eat? OR eating OR ate OR intak* OR purchas* OR buy* OR demand* OR choos* OR choice* OR avoid* OR prefer*)).ab,ti

2.2. Embase (Elsevier)

('animal welfare'/exp OR 'emotion'/de OR 'disgust'/de OR 'fear'/de OR 'guilt'/de OR 'empathy'/exp OR 'morality'/exp OR (emotion* OR affective OR disgust* OR fear OR guilt* OR empath* OR suffer* OR moral* OR ethic* OR humane* OR anthropomorph* OR belief* OR 'cognitive dissonan*' OR 'meat paradox'):ab,ti OR (animal* NEAR/6 (welfare OR rights OR wellbeing OR 'well being' OR cruel* OR abuse OR mistreat*)):ab,ti) AND ('meat consumption'/exp OR ((meat* OR 'animal product*' OR beef OR veal OR lamb OR pork OR poultry OR chicken OR turkey OR pig OR cow OR sheep) NEAR/6 (consum* OR eat OR eats OR eating OR ate OR intak* OR purchas* OR buy* OR demand* OR choos* OR choice* OR avoid* OR prefer*)):ab,ti)

2.3. Web of Science (Clarivate Analytics)

TS=("emotion*" OR "affective" OR "disgust*" OR "fear" OR "guilt*" OR "empath*" OR "suffer*" OR "moral*" OR "ethic*" OR "humane*" OR "anthropomorph*" OR "belief*" OR "cognitive dissonan*" OR "meat paradox" OR ("animal*" NEAR/6 ("welfare" OR "rights" OR "wellbeing" OR "well being" OR "cruel*" OR "abuse" OR "mistreat*"))) AND TS=(("meat*" OR "animal product*" OR "beef" OR "veal" OR "lamb" OR "pork" OR "poultry" OR "chicken" OR "turkey" OR "pig" OR "cow" OR "sheep") NEAR/6 ("consum*" OR "eat" OR "eats" OR "eating" OR "ate" OR "intak*" OR "purchas*" OR "buy*" OR "demand*" OR "choos*" OR "choice*" OR "avoid*" OR "prefer*"))

2.4. PsycINFO (EBSCO)

(DE ("Animal Welfare" OR "Animal Cruelty" OR "Animal Rights" OR "Emotional Content" OR "Emotional Responses" OR "Emotions" OR "Disgust" OR "Fear" OR "Guilt" OR "Empathy" OR "Morality" OR "Cognitive Dissonance") OR TI (emotion* OR affective OR disgust* OR fear OR guilt* OR empath* OR suffer* OR moral* OR ethic* OR humane* OR anthropomorph* OR belief* OR "cognitive dissonan*" OR "meat paradox" OR (animal* N6 (welfare OR rights OR wellbeing OR "well being" OR cruel* OR abuse OR mistreat*))) OR AB (emotion* OR affective OR disgust* OR fear OR guilt* OR empath* OR suffer* OR moral* OR ethic* OR humane* OR anthropomorph* OR belief* OR "cognitive dissonan*" OR "meat paradox" OR (animal* N6 (welfare OR rights OR wellbeing OR "well being" OR cruel* OR abuse OR mistreat*)))) AND (TI ((meat* OR "animal product*" OR beef OR veal OR lamb OR pork OR poultry OR chicken OR turkey OR pig OR cow OR sheep) N6 (consum* OR eat OR eats OR eating OR ate OR intak* OR purchas* OR buy* OR demand* OR choos* OR choice* OR avoid* OR prefer*)) OR AB ((meat* OR "animal product*" OR beef OR veal OR lamb OR pork OR poultry OR chicken OR turkey OR pig OR cow OR

sheep) N6 (consum* OR eat OR eats OR eating OR ate OR intak* OR purchas* OR buy* OR demand* OR choos* OR choice* OR avoid* OR prefer*)))

2.5. CAB Abstracts (Clarivate Analytics)

TS=("emotion*" OR "affective" OR "disgust*" OR "fear" OR "guilt*" OR "empath*" OR "suffer*" OR "moral*" OR "ethic*" OR "humane*" OR "anthropomorph*" OR "belief*" OR "cognitive dissonan*" OR "meat paradox" OR ("animal*" NEAR/6 ("welfare" OR "rights" OR "wellbeing" OR "well being" OR "cruel*" OR "abuse" OR "mistreat*")))) AND TS=(("meat*" OR "animal product*" OR "beef" OR "veal" OR "lamb" OR "pork" OR "poultry" OR "chicken" OR "turkey" OR "pig" OR "cow" OR "sheep") NEAR/6 ("consum*" OR "eat" OR "eats" OR "eating" OR "ate" OR "intak*" OR "purchas*" OR "buy*" OR "demand*" OR "choos*" OR "choice*" OR "avoid*" OR "prefer*"))

2.6. Sociological Abstracts (ProQuest)

(SU.EXACT("Animal Human Relations" OR "Emotions" OR "Guilt" OR "Fear" OR "Empathy" OR "Morality" OR "Moral Judgement" OR "Cognitive Disonance") OR TI(emotion* OR affective OR disgust* OR fear OR guilt* OR empath* OR suffer* OR moral* OR ethic* OR humane* OR anthropomorph* OR belief* OR "cognitive dissonan*" OR "meat paradox" OR (animal* NEAR/6 (welfare OR rights OR wellbeing OR "well being" OR cruel* OR abuse OR mistreat*))) OR AB(emotion* OR affective OR disgust* OR fear OR guilt* OR empath* OR suffer* OR moral* OR ethic* OR humane* OR anthropomorph* OR belief* OR "cognitive dissonan*" OR "meat paradox" OR (animal* NEAR/6 (welfare OR rights OR wellbeing OR "well being" OR cruel* OR abuse OR mistreat*)))) AND ((meat* OR "animal product*" OR beef OR veal OR lamb OR pork OR poultry OR chicken OR turkey OR pig OR cow OR sheep) NEAR/6 (consum* OR eat OR eats OR eating OR ate OR intak* OR purchas* OR buy* OR demand* OR choos* OR choice* OR avoid* OR prefer*))

2.7. ProQuest Dissertations and Theses (ProQuest)

noft(((meat* OR "animal product*" OR beef OR veal OR lamb OR pork OR poultry OR chicken OR turkey OR pig OR cow OR sheep) NEAR/6 (consum* OR eat OR eats OR eating OR ate OR intak* OR purchas* OR buy* OR demand* OR choos* OR choice* OR avoid* OR prefer*))) AND noft((emotion* OR affective OR disgust* OR fear OR guilt* OR empath* OR suffer* OR moral* OR ethic* OR humane* OR anthropomorph* OR belief* OR "cognitive dissonan*" OR "meat paradox" OR (animal* NEAR/6 (welfare OR rights OR wellbeing OR "well being" OR cruel* OR abuse OR mistreat*))))

2.8. PolicyFile (ProQuest)

(((meat* OR "animal product*" OR beef OR veal OR lamb OR pork OR poultry OR chicken OR turkey OR pig OR cow OR sheep) NEAR/6 (consum* OR eat OR eats OR eating OR ate OR intak* OR purchas* OR buy* OR demand* OR choos* OR choice* OR avoid* OR prefer*))) AND noft((emotion* OR affective OR disgust* OR fear OR guilt* OR empath* OR

suffer* OR moral* OR ethic* OR humane* OR anthropomorph* OR belief* OR "cognitive dissonan*" OR "meat paradox" OR (animal* NEAR/6 (welfare OR rights OR wellbeing OR "well being" OR cruel* OR abuse OR mistreat*))))

3. Examples of websites to be searched for grey literature

- Faunalytics (https://faunalytics.org/library/)
- Animal Welfare Action Lab (http://www.awalab.org/)
- The Humane League Labs (http://www.humaneleaguelabs.org/reports/)
- Mercy for Animals (https://mercyforanimals.org/research)
- Animal Charity Evaluators (https://animalcharityevaluators.org/research/)
- Better Buying Lab (https://www.wri.org/our-work/project/better-buying-lab/research-and-resources)

4. Details of data extraction

To ensure that point estimates are statistically and conceptually comparable across studies, we will extract, when feasible, point estimates for each study that represent the risk ratio of "low" versus "high" meat consumption or purchase. For studies reporting sufficient information or with raw data available, we will define "low" and "high" relative to the median consumption or purchasing outcome in the control condition (i.e., "high" consumption for a two-group randomized trial would be defined as consumption higher than the median consumption in the control group). Dichotomizing at the median, rather than at a more extreme value, improves statistical precision. For longitudinal studies, we will estimate the risk ratio controlling for subjects' baseline consumption or purchase to increase statistical precision. Some studies may define the outcome measure not in absolute terms (e.g., servings consumed) but in relative terms (e.g., reducing meat consumption from one's previous consumption), in which case we will use the risk ratio representing reducing (versus maintaining or increasing) consumption or purchase. For studies that do not report information sufficient to extract the above risk ratios, and for which raw data are not available for re-analysis, we will instead attempt to extract or calculate a risk ratio using the studies' own criteria for "low" and "high" meat consumption if such a dichotomy was used. However, if the study treats consumption or purchase as continuous variables, we will extract or calculate a point estimate on the standardized mean difference scale and then approximately convert the estimate to the risk ratio scale[1;2].

References

[1] Susan Chinn. A simple method for converting an odds ratio to effect size for use in meta-analysis. *Statistics in Medicine*, 19(22):3127–3131, 2000.

[2] Tyler VanderWeele. On a square-root transformation of the odds ratio for a common outcome. *Epidemiology (Cambridge, Mass.)*, 28(6):e58, 2017.
